# 18-month occurrence of severe events among early diagnosed HIV-infected children before antiretroviral therapy in Abidjan, Côte d'Ivoire: A cohort study

**DOI:** 10.1186/1471-2458-8-169

**Published:** 2008-05-20

**Authors:** Jérôme Harambat, Patricia Fassinou, Renaud Becquet, Pety Touré, François Rouet, François Dabis, Philippe Msellati, Stéphane Blanche, Marguerite Timité-Konan, Roger Salamon, Valériane Leroy, ANRS 1201/1202 Ditrame Plus Study Group

**Affiliations:** 1 INSERM, Unité 897, Bordeaux, France; 2 Institut de Santé Publique, Epidémiologie et Développement (ISPED), Université Victor Segalen, Bordeaux 2, France; 3 Projet ANRS 1201/1202 Ditrame Plus, Programme PAC-CI, Centre Hospitalier Universitaire de Treichville, Abidjan, Côte d'Ivoire; 4 Service de Pédiatrie, Centre Hospitalier Universitaire de Yopougon, Abidjan, Côte d'Ivoire; 5 Centre de Diagnostic et de Recherches sur le SIDA (CeDReS), Centre Hospitalier Universitaire de Treichville, Abidjan, Côte d'Ivoire; 6 UMR 145, Institut de Recherche pour le Développement, Montpellier, France; 7 Service de Pédiatrie, Centre Hospitalier Universitaire Necker Enfants Malades, Paris, France; 8 See appendix

## Abstract

**Objective:**

To assess the 18-month field effectiveness on severe events of a pediatric package combining early HIV-diagnosis and targeted cotrimoxazole prophylaxis in HIV-infected children from age six-week before the antiretroviral era, in Abidjan, Côte d'Ivoire.

**Methods:**

Data from two consecutive prevention of HIV mother-to-child transmission programs were compared: the ANRS 1201/1202 Ditrame-Plus cohort (2001–2005) and the pooled data of the ANRS 049a Ditrame randomized trial and its following open-labeled cohort (1995–2000), used as a reference group. HIV-infected pregnant women ≥ 32–36 weeks of gestation were offered a short-course peri-partum antiretroviral prophylaxis (ZDV in Ditrame, and ZDV ± 3TC+single-dose (sd) NVP in Ditrame-Plus). Neonatal prophylaxis was provided in Ditrame-Plus only: 7-day ZDV and sdNVP 48–72 h after birth. A 6-week pediatric HIV-RNA diagnosis was provided on-line in the Ditrame-Plus while it was only oriented on clinical symptoms in Ditrame. Six-week HIV-infected children received a daily cotrimoxazole prophylaxis in Ditrame-Plus while no prophylaxis was provided in Ditrame. The determinants of severe events (death or hospitalization > 1 day) were assessed in a Cox regression model.

**Results:**

Between 1995 and 2003, 98 out of the 1121 live-births were diagnosed as HIV-infected in peri-partum: 45 from Ditrame-Plus and 53 from Ditrame. The 18-month Kaplan-Meier cumulative probability of presenting a severe event was 66% in Ditrame-Plus (95% confidence interval [95%CI]: 50%–81%) and 77% in Ditrame (95%CI: 65%–89%), Log Rank test: p = 0.47. After adjustment on maternal WHO clinical stage, maternal death, 6-week pediatric viral load, birth-weight, and breastfeeding exposure, the 18-month risk of severe event was lower in Ditrame-Plus than in Ditrame (adjusted Hazard Ratio (aHR): 0.55, 95%CI: 0.3–1.1), although the difference was not statistically significant; p = 0.07). Maternal death was the only variable determinant of the occurrence of severe events in children (aHR: 3.73; CI: 2.2–11.2; p = 0.01).

**Conclusion:**

Early cotrimoxazole from 6 weeks of age in HIV-infected infants seemed to reduce probability of severe events but the study lacked statistical power to prove this. Even with systematic cotrimoxazole prophylaxis, infant morbidity and mortality remained high pointing towards a need for early pediatric HIV-diagnosis and antiretroviral treatment in Africa.

## Background

Randomized clinical trials have shown the efficacy of short antiretroviral regimens in preventing mother to child transmission of HIV (PMTCT) in Africa [1,2]. Despite these encouraging findings, less than 11% of HIV-infected pregnant women had received one of these PMTCT interventions in low-income countries in 2005 [3]. Therefore, between 570,000 and 740,000 children became infected with HIV in 2005 worldwide. In 2005, 750,000 children died of AIDS-related diseases of whom 87% were living in sub-Saharan Africa and most had been infected by mother-to-child transmission (MTCT) [4].

It is now urgent to improve the neglected management of HIV-infected children to reduce paediatric mortality in Africa. Indeed, child mortality related to HIV/AIDS is high and occurs early in Africa because of the rapid development of disease manifestations within the first two years of life [5]. Mortality rates were estimated to be 35.2% by age 1 year and 52.5% by age 2 year among HIV-infected children in a recent African meta-analysis. As in industrialized countries before the antiretroviral era, the paediatric HIV disease seems to follow a bimodal course in Africa [6,7]. Thus, those children who survive beyond two years might have a slightly slower disease progression [6,7].

In Côte d'Ivoire, the overall infant mortality rate was estimated to be around 118 out of 1,000 live-births in 1998 [8]. While in Abidjan, half of HIV-infected children died before their first birthday in the absence of highly active antiretroviral therapy [9]. Most of these early deaths were attributable to infectious diseases potentially preventable by cotrimoxazole [9-11]. We therefore hypothesised that an early prophylaxis by cotrimoxazole would help HIV-infected children in surviving beyond their first birthday so they would have a chance to become eligible for a specific paediatric antiretroviral therapy later on.

The benefit of cotrimoxazole is already well established in HIV-infected adults [12-14]. In children mainly older than 12 months, a trial has shown a significant benefit of cotrimoxazole prophylaxis in HIV-infected children aged 1–14 years in Zambia [15]. Since 2000, cotrimoxazole prophylaxis is recommended in HIV-exposed children [16,17]. However, its field efficacy within the first year of life is unknown in Africa and needs to be balanced with the safety issue.

Within the ANRS 1201/1202 Ditrame-Plus cohort conducted in Abidjan, Côte d'Ivoire, infants born to HIV-infected mothers who received an PMTCT intervention [18,19] were offered a paediatric package including a routine and early diagnosis of HIV infection from age 6-week by real-time PCR [20,21] and a cotrimoxazole prophylaxis targeted on HIV-infected infants from then.

The objective of this study was to assess the field effectiveness of this early paediatric package on the occurrence of severe events during the first 18 months the ANRS 1201/1202 Ditrame-Plus cohort conducted in 2001–2005 [18,19] and compared it with a historical cohort of children followed-up in the 1995–2000 period as a reference group. in Abidjan, Côte d'Ivoire [9].

## Methods

### Study design

For this study, we compared data issued from the ANRS 1201/1202 Ditrame-Plus cohort [18,19] and the pooled data of the ANRS 049a Ditrame randomized clinical trial and its following open-labelled cohort from 1995 to 2000 [22-24]. During the 1995–2000 and 2001–2005 periods, the paediatric follow-up was conducted in the same sites and by the same clinical team following the same schedule. These projects were approved by the National Ethical Committee in Côte d'Ivoire and the institutional review board of the French Agence Nationale de Recherches sur le Sida (ANRS).

### Population and peri-partum PMTCT intervention

The inclusion procedures and research design undertaken in these two projects were previously described [18,19,22,23]. Briefly, any consenting HIV-1 infected pregnant woman aged 18 years and over, at < 32–36 weeks of gestation (and 34–36 for Ditrame), in one of the selected community-run health facilities was eligible. Women included were systematically proposed a peri-partum PMTCT antiretroviral regimen (Additional file 1: Table 1). The maternal antiretroviral prophylaxis consisted of a short-course peripartum zidovudine (ZDV) regimen in the ANRS 049a DITRAME trial and a combination of ZDV ± lamivudine (3TC) and nevirapine single dose (NVPsd) in the Ditrame-Plus study. Neonatal prophylaxis in the Ditrame-Plus study consisted of ZDV for 7 days and a sdNVP 48–72 h after birth.

### Postnatal PMTCT intervention

No specific postnatal intervention was provided in the ANRS 049a Ditrame trial (Additional file 1: Table 1). At inclusion in the ANRS 1201/1202 Ditrame-Plus cohort, pregnant women were systematically offered two alternative options to prolonged breastfeeding: exclusive formula-feeding from birth (with a drug inhibiting lactation) or exclusive breastfeeding with early cessation within the fourth month (short-term breastfeeding) [25,26]. The staff supported their choice and counselled them accordingly. Breastmilk substitutes were free of charge from birth or the date of weaning until nine months of age. A systematic vitamin A supplementation was provided to children according to WHO recommendations.

### Paediatric diagnosis of HIV infection

HIV diagnosis was provided at 6-week on-line in the Ditrame-Plus project while it was only oriented on clinical symptoms in the Ditrame study. Blood samples were taken for paediatric HIV-1 diagnosis at Day 2, week 4–6, then three monthly until one year, month 18 and 24 and two months after complete cessation of breastfeeding if any. A quantitative bDNA assay (Quantiplex HIV RNA 3.0; Quantiplex, East Walpole, Massachussets, USA) was retrospectively applied to the 1995–2000 cohort [20]. In Ditrame Plus, a paediatric HIV-1 infection was initially diagnosed using a commercial plasma HIV-1 RNA assay (Versant bDNA HIV RNA kit version 3.0, Bayer diagnostics, Emeryville, CA, USA) [20]. From 2003, a TaqMan HIV-1 RNA real-time PCR test was used [21].

Paediatric HIV-1 infection was defined as a positive HIV RNA at any age, or as a positive HIV serology if age > 18 months. The first positive test allowed the estimation of the timing of infection: in utero if the Day-2 sample was positive, intrapartum-early postnatal if the Day-2 sample was negative but the 4–6-week sample positive, late postnatal if the 4–6-week sample was negative but later sample became positive.

### Cotrimoxazole prophylaxis

In the 1995–2000 period, no prophylaxis of opportunistic infections was provided in the absence of Ivoirian guidelines. After a national consensus meeting held in 1999 in Abidjan, Côte d'Ivoire and an international WHO/UNAIDS meeting held in 2000 in Harare, Zimbabwe, we applied the recommendations formulating that cotrimoxazole prophylaxis should be systematically used in HIV-infected children and in HIV-exposed infants until diagnosis of its own infection has been made [16,27]. From 2001, HIV-infected children received a cotrimoxazole prophylaxis (25 mg/kg per day) systematically from six weeks of age until at least their first birthday.

### Follow-up procedures and data collection

From birth up to 18 months, systematic visits were scheduled on study sites for clinical, psychosocial and biological follow-up for both mothers and infants. In both PMTCT programs, children were seen at birth, day 2, then at week-1, week-6, month-3 then three monthly until 18 months. At each contact, the medical staff documented clinical events that occurred in children since the last visit and infant feeding practices were recorded. During the Ditrame-Plus study, children were first managed at the day-care hospital units linked to the centres, then transferred to the University Hospital of Yopougon if overnight care were needed. In Ditrame, there was no day-care hospital. In both cohorts, children were referred to the paediatric unit of the University Hospital of Yopougon for life-threatening diseases or diseases requiring overnight care (> one day). Care services were also available whenever needed between scheduled visits. All transport costs were reimbursed and all care expenses were entirely supported by both projects.

### Study outcomes

The primary outcome of the study was the occurrence of a severe event defined as death or a hospitalisation (> one day) related to any cause in the paediatric unit of the University Hospital. Mortality was also investigated separately as a secondary outcome. Causes of death were explored in all children. Verbal autopsies were systematically conducted by trained socio-psychologists to assign a possible cause of death in Ditrame-Plus. Probable contributing causes of death were independently assessed by two paediatricians on the basis of all the clinical information collected (including hospital records) and the verbal autopsy. A third evaluation was provided in case of disagreement. Clinical and haematological incidence of adverse events graded 3 or 4 (WHO standardised toxicity table) of cotrimoxazole were looked for systematically at each visit in Ditrame-Plus.

### Statistical analysis

All live-born infants infected with HIV in peri-partum were included in this analysis. Late postnatal cases were not eligible for an early cotrimoxazole prophylaxis and excluded from the present analysis.

Baseline characteristics were compared between the Ditrame-Plus cohort and the Ditrame historical control cohort using Pearson χ^2 ^test or Fisher's exact test for categorical variables, and Student's *t*-test or Mann-Whitney U test for continuous variables. Maternal CD4 results were expressed as percentage of total lymphocyte count, maternal and paediatric plasma viral load were expressed in log_10_/mL. The week-6 paediatric plasma viral load estimate was used as considered the highest level for children infected in peripartum [28]. In Ditrame Plus, the allocation to an infant-feeding group (formula-fed or short-term breastfed) was based on the actual feeding option implemented two days after birth. In Ditrame, prolonged and unrestricted breastfeeding was the norm [29].

The cumulative probability of having a first severe event (hospitalization > one day or death, which ever came first) from birth was assessed using a Kaplan-Meier estimation with a time-to-failure method. The date of right censoring was the date of antiretroviral therapy introduction, or the last available date of follow-up until 18 months. An adjusted Cox proportional-hazard model was used to study the determinants of the 18-month occurrence of severe events, including the ANRS 1201/1202 Ditrame-Plus paediatric package. Hazard Ratio (HR) for severe events between the two groups was estimated with adjustment for other known determinants of child mortality and other potential confounding factors at baseline. HRs were reported with their 95% confidence interval (95%CI). The role of interaction terms was also investigated. All statistical analyses were carried out with the use of SAS software (version 8.2; SAS Institute, Cary, North Carolina, USA).

## Results

### Study population

**Figure 1:**
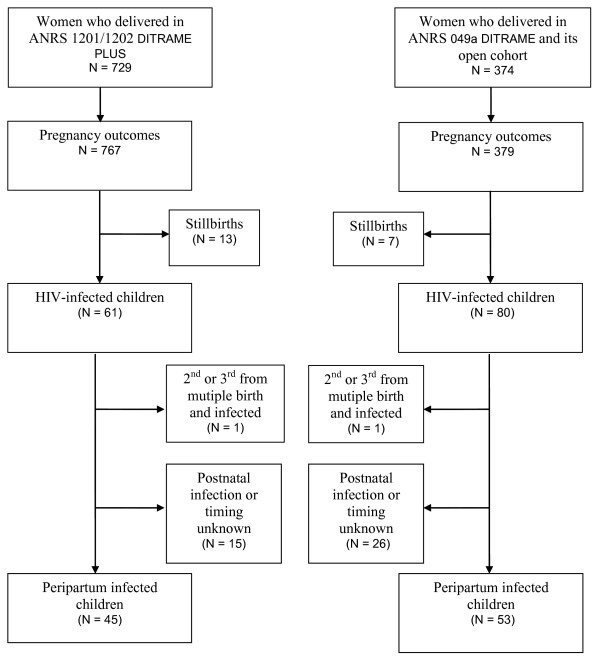
**Cohort profile of children included in the ANRS 049a DITRAME trial and its open cohort and the ANRS 1201/1202 DITRAME-PLUS cohort.** 1995–2005.

Out of the 1164 HIV-infected pregnant women included between September 1995 and July 2003, 1103 (729 from Ditrame Plus and 374 from Ditrame) gave birth to 1121 live-births. Of these, 141 were diagnosed as HIV-infected during the first 18 months follow-up (Figure 1). After exclusion of the late postnatal transmission cases and the second and third born babies of multiple birth outcomes, 98 peripartum HIV-infected children were included in the present analysis: 45 were issued from the Ditrame-Plus project (25 exposed to maternal ZDV+NVPsd and 20 to ZDV+3TC+NVPsd) and 53 constituted the historical control group (23 exposed to maternal placebo before 1998, and 30 to maternal ZDV).

### Baseline and follow-up characteristics

Before delivery, mothers of HIV-infected children from the Ditrame-Plus cohort were significantly more advanced in HIV-disease than those from the Ditrame control cohort (Additional file 1: Table 2): they were more likely classified WHO clinical stage 3–4, more immune-suppressed (median difference: -165 CD4+ cell count) and had a higher viral load (mean difference: +0.3 log). Twenty children (44.4%) were formula-fed from birth in the Ditrame-Plus cohort and only two (3.8%) in Ditrame. HIV-infected children in the Ditrame-Plus cohort were significantly more likely to have been infected during the in-utero period than children in the Ditrame trial, 53.3% and 11.3%, respectively (p < 0.01). The median baseline 6-week paediatric plasma viral load at HIV-diagnosis was significantly higher in Ditrame-Plus compared to Ditrame, with a mean difference of 0.4 log. There was no statistical difference between the two groups for other socio-demographic, clinical and biological baseline characteristics. Among the 45 HIV-infected children from Ditrame-Plus, 31 (69%) had at least one CD4+ cell percentage measurement at a median age 3-month. Their median CD4 percentage was 18% (range: 6%–37%).

The median follow-up duration from birth was 12.1 months among Ditrame-Plus children and 8.7 months in the Ditrame trial (p = 0.13). Two children (4.4%) and five children (9.4%) were lost-to-follow-up by age 18-month in the Ditrame-Plus cohort and the Ditrame trial respectively. Nine of the 45 Ditrame-Plus children (20%) received an antiretroviral therapy initiated at a median age of 5.5 months and were right-censured whereas none were treated before age 18-month in Ditrame. Over the first 18-months, five maternal deaths were recorded of whom four occurred in the Ditrame-Plus cohort.

### Occurrence of severe events

**Figure 2:**
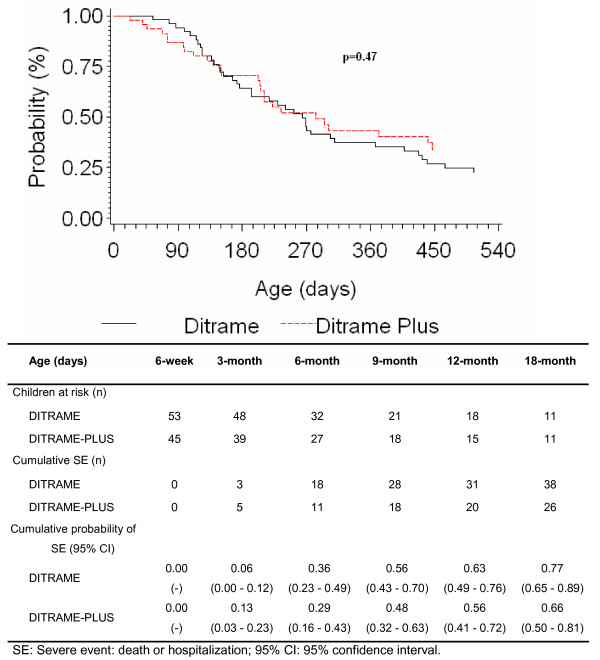
**Kaplan-Meier probability of 18-month occurrence of severe events (hospitalization > one day or death) in 98 peri-partum HIV-children children in the ANRS 049a DITRAME and ANRS 1201/1202 DITRAME PLUS projects.** Abidjan, Côte d'Ivoire. 1995–2005.

Among the 98 HIV-infected children, 64 severe events occurred during the first 18 months of life: 26 in the 45 Ditrame-Plus children and 38 out of the 53 Ditrame-children. The Kaplan-Meier cumulative probabilities of presenting a severe event did not differ significantly between the two cohorts (Figure 2, Log Rank, p = 0.47): at 12-month, 56% (95%CI: 41%–72%) in Ditrame-Plus and 63% (95%CI: 49%–76%) in the control group; and at 18-month, 66% (95%CI: 50%–81%) in Ditrame-Plus and 77% (95%CI: 65%–89%) in the control group. After adjustment on maternal WHO clinical staging, maternal death as a time dependant variable, 6-week paediatric viral load, low birth-weight, and breastfeeding exposure, the risk of severe event over the first 18 months tended to be lower in Ditrame-Plus than in Ditrame, but this trend did not reach the statistical difference (aHR: 0.55; 95%CI: 0.3–1.1; p = 0.07). Maternal death as time dependant variable was the only variable significantly associated with the occurrence of severe events in children (aHR: 3.73; CI: 2.2–11.2; p = 0.01) (Additional file 1: Table 3).

### Mortality

Overall, there were 23 deaths out of the 45 children from the Ditrame-Plus cohort and 34 out of the 53 in the Ditrame group. Survival did not differ significantly between these two groups of HIV-infected children: the 12-month Kaplan-Meier probability of death were 50% (95%CI: 34%–66%) in Ditrame-Plus and 59% (95%CI: 45%–72%) in the control group; at 18-month, they were 60% (95%CI: 44%–77%) in Ditrame-Plus and 69% (95%CI: 56%–82%) in Ditrame (Log Rank, p = 0.49). Primary causes of death tended to differ between the two cohorts: Diarrhoea was the main cause of mortality in Ditrame-Plus (43%) and was more often reported than in controls (p = 0.03), while the leading cause of death reported in Ditrame was pneumonia (41%). Other causes of death were comparable between the two cohorts (Additional file 1: Table 4).

### Safety

Among the 45 HIV-infected children who received cotrimoxazole in Ditrame-Plus, no clinical grade 3 or 4 adverse event (dermatologic or Lyell Syndrome) attributable to cotrimoxazole was reported. The incidence rate of severe neutropenia (< 1000/mm^3^) was 20 per 100 child-years of cotrimoxazole follow-up (N = 8). The incidence rate of grade 3 or 4 thrombocytopenia (< 100 000/mm^3^) and anaemia (< 8 g/dL) were 28 (N = 11) and 51 (N = 14) per 100 child-years respectively. Among the 12 blood cultures available, 50% found germs resistant to cotrimoxazole.

## Discussion

We compared pooled data collected over two successive west-African PMTCT cohorts conducted in a similar environment to assess the field effectiveness of a specific paediatric set of interventions combining early HIV-diagnosis in all HIV-exposed newborns from age 6-week and cotrimoxazole prophylaxis targeted to those HIV-infected. Early virological HIV-diagnosis was routinely feasible in Ditrame-Plus, and despite the systematic daily cotrimoxozole prophylaxis, the occurrence of severe events remained extremely high reaching 66% at 18 months of age. These residual severe events remained also mainly attributable to infectious causes. After adjustment on known determinants of child morbidity-mortality, the daily cotrimoxazole provided to HIV-infected children was well tolerated and reduced by 45%, although not significantly, the occurrence of severe morbidity-mortality when compared to the control group. The effectiveness of cotrimoxazole prophylaxis for optimizing early case management of HIV-infected infants and reducing severe morbidity-mortality within their first year of life was not statistically significant within our study. In fact, the statistical power estimated to show an existing difference between the two cohorts was estimated to be only 11%.

As the access to care was different between the two cohorts with no day-care hospital in Ditrame while this existed in the Ditrame Plus cohort, .some of the Ditrame plus children were first managed at the day-care hospital before going back to home on the same day or to be hospitalised at the pediatric unit if overnight care was needed. To get the most comparable definition of hospitalisation between the two cohorts, we defined hospitalisation as a hospitalisation > one day, at the pediatric unit of the Yopougon Hospital for both cohorts. Consequently, we feel that we have minimized the potential information bias in documenting comparably the risk of severe event.

The 6-week MTCT rates were about 15% in the Ditrame trial [30] while it was 5% in the Ditrame-Plus cohort, explained by a greater efficacy of PMTCT interventions in Ditrame-Plus [18] but also leading to the likely selection of HIV-infected newborns at higher risk of rapid disease progression. At inclusion in Ditrame-Plus, mothers from HIV-infected children were more advanced in the HIV/AIDS disease, more immune-suppressed, and their infected children were more likely to have been infected earlier (in utero). Therefore, we observed a trend of a protective effect of cotrimoxazole in Ditrame-Plus HIV-infected children having a poorer prognosis than children from Ditrame.

In the Ditrame-plus cohort, 20% of the children started an antiretroviral therapy as they were symptomatics, with a likely higher risk of experiencing a severe event: if these children would have not been right censored at the time of starting antiretroviral therapy in our analysis, this would have led to a probably lower risk of severe event in the ditrame plus cohort compared to the ditrame cohort but attributable to the ART effect rather than the cotrimoxazole effect, so with an overestimation of the cotrimoxazole effect.

We conclude that we have underestimated the true cotrimoxazole protective effect: we hypothesize the cotrimoxazole protective effect would have in fact probably exist with a greater public health impact if it was administrated to HIV-infected infants with a less severe HIV disease than in Ditrame-Plus and issued from HIV-infected mothers who would have received a less effective PMTCT intervention than the Ditrame-Plus antiretroviral combination. So, this would even have a greater impact in population with a lower standard of care access than in our well structured research context.

The ideal study design to assess the efficacy of cotrimoxazole prophylaxis given early in newborn would have been a randomized placebo controlled trial. Despite the lack of evidence of cotrimoxazole's effectiveness specifically in African infants less than 12 months in the CHAP trial [15], this would have been unethical because of the lack of equipoise between the two randomised cotrimoxazole and placebo arms as cotrimoxazole was already demondtrated to be efficacious in children older than 12 months [15] and in adults [13]. Based on these latter finding, the good clinical practice was to provide the recommended standard of care to all study participants: national guidelines recommend cotrimoxazole prophylaxis in children since 1999 in Côte d'Ivoire [27]. We choose this historical comparison to assess the cotrimoxazole effectiveness adjusting for the other known prognosis factors of infant mortality. We acknowledge that although important confounding variables such as maternal clinical stage of disease, 6-week paediatric viral load, birth-weight, breastfeeding were systematically controlled for in the analysis, this potential confusion was only partly taken into account notably because infant CD4 cell count was not available in the Ditrame study. Finally, the low rates of lost-to-follow-up of these HIV-infected children were acceptable in this difficult context and strengthened our conclusion.

The infant mortality figures among HIV-infected children reported in the Ditrame and Ditrame-Plus projects (59% and 50% respectively) were higher than those reported in a pooled analysis in which 35% of African children with early HIV-infection had died by age one year [5]. The difference could be due to the large proportion of South African children in this pooled analysis, where infant mortality rate was reported to be lower than in West Africa according to 1998 demographic health surveys [5].: i.e. 59 per 1,000 livebirths in South Africa [31] versus 118 per 1,000 in Côte d'Ivoire [8]. Our results suggest a reduced incidence of pneumonia as cause of death but a significant higher rate of diarrhoea as cause of death was observed in Ditrame-Plus compared to Ditrame. About 40% of the Ditrame-Plus children were formula-fed while their HIV-status was not yet ascertained, exposing formula-fed HIV-infected children to an increased mortality risk. Formula-feeding was not retained as a significant explaining factor for morbidity-mortality in our adjusted analysis, but HIV-infected children who are still breastfed at the time of HIV-diagnosis should better continue to be breastfed as recently reported elsewhere [32] and recommended [33].

As in others studies, our results confirm maternal death as the major determinant of infant mortality suggesting the crucial role of caregivers including mothers in the access to care for children in Africa [5,34]. These findings thus emphasise the need for an early HIV-diagnosis, and an adequate access to antiretroviral care and support for HIV-infected children and all members of affected families, including mothers [3].

Cotrimoxazole demonstrated its efficacy in 2004 in a Zambian trial conducted among children mainly older than 12 months [15]. In two South African studies, the history of cotrimoxazole use was significantly lower in children with severe pneumonia attributable to *Pneumocystis jiroveci *than those without [35,36]. Lastly, HIV-infected infants with access to cotrimoxazole prophylaxis had a significantly lower incidence of pneumonia than those without access to prophylaxis [37]. Our results are consistent with the Zambian and South African results observed in older children and reinforce the message suggesting that all HIV-infected children should receive cotrimoxazole prophylaxis in low-income settings, irrespective of their age. In 2006, WHO recommends that all infants exposed to HIV should receive cotrimoxazole prophylaxis from six weeks until HIV infection is excluded (rarely before 18 months in routine) [17].

While the number of antiretroviral-treated adults in low-income countries is increasing in Africa with the World Health Organization (WHO) 3 × 5 initiative since 2003, it is estimated that only 15% of people in need of antiretroviral treatment in 2005 are covered [38]. There is particularly a lack of access to antiretroviral therapy in African children [3]. The lack of routine available access to an early diagnosis of HIV-infection in children may explain this delayed access to antiretroviral therapy [39]. In our setting, the real-time PCR test used was low cost (10 euros/sample) and highly accurate [21]. The cost of providing systematically a daily cotrimoxazole prophylaxis to any HIV-exposed child (3 euros/month) during their first year of life is estimated to be (100*10 months*3) = 3000 euros for 100 HIV-exposed children. When using a targeted strategy guided by the early paediatric HIV-diagnosis (10 euros per unit), given the hypothesis of a 5% mother-to-child transmission rate, the cost for diagnosing 100 HIV-exposed children and providing to HIV-infected children a daily cotrimoxazole prophylaxis during their first year of life is estimated to be [(100*10)+(5*10 months*3)] = 1150 euros for 100 HIV-exposed children. Thus, not only is the cost of the targeted strategy more affordable, but also will it allow detecting early HIV-infected children to give them an opportunity to access to antiretroviral therapy.

## Conclusion

To conclude, early cotrimoxazole from 6 weeks of age in HIV-infected infants seemed to reduce probability of severe events but the study lacked statistical power to prove this. Even with systematic cotrimoxazole prophylaxis, infant morbidity and mortality remained high. Our results highlight the expected interest of early antiretroviral therapy in HIV-infected children less than 12 months of age, as this was already reported from several studies conducted in developed countries [40-42]. This early antiretroviral therapy has recently demonstrated a benefit on infant survival in South Africa [43]. Our results argued that an early and reliable pediatric HIV-diagnosis from age 6-week could better reduce the burden of pediatric HIV-infection in giving a universal opportunity of access to cotrimoxazole prophylaxis and antiretroviral therapy for them and their family.

## Appendix

### Composition of the ANRS 1201/1202 Ditrame-Plus Study Group

Principal Investigators: François Dabis, Valériane Leroy, Marguerite Timite-Konan, Christiane Welffens-Ekra. Coordination in Abidjan: Laurence Bequet, Didier K. Ekouévi, Besigin Tonwe-Gold, Ida Viho. Methodology, biostatistics and data management: Gérard Allou, Renaud Becquet, Katia Castetbon, Laurence Dequae-Merchadou, Charlotte Sakarovitch, Dominique Touchard. Clinical team: Clarisse Amani-Bosse, Ignace Ayekoe, Gédéon Bédikou, Nacoumba Coulibaly, Christine Danel, Patricia Fassinou, Apollinaire Horo, Ruffin Likikouët, Hassan Toure. Laboratory team: André Inwoley, François Rouet, Ramata Touré. Psycho-social team: Hortense Aka-Dago, Alphonse Sihé. Social-sciences team: Hélène Agbo, Hermann Brou, Annabel Desgrées-du-Loû, Annick Tijou-Traoré, Benjamin Zanou. Scientific Committee: Stéphane Blanche, Jean-François Delfraissy, Philippe Lepage, Laurent Mandelbrot, Christine Rouzioux, Roger Salamon.

#### Ethical permissions

The ANRS 1201/1202 Ditrame-Plus study was granted ethical permission in Côte d'Ivoire from the ethical committee of the National AIDS Control Programme, and in France from the institutional review board of the French Agence Nationale de Recherches sur le Sida (ANRS).

#### Sponsorship

The primary sponsor of the ANRS 049a trial and the ANRS 1201/1202 Ditrame-Plus Cohort was the Agence Nationale de Recherches sur le Sida (ANRS). Jérôme Harambat was a fellow of the French Foundation for Medical Research during his master of public health. Renaud Becquet was a fellow of the French Ministry of Education, Research and Technology and is now a post-doctoral fellow of the French charity SIDACTION. François Rouet was supported by the French Ministry of Foreign Affairs.

## Competing interests

The authors declare that they have no competing interest.

## Authors' contributions

JH carried out the statistical analysis, interpreted the results, and contributed to the writing manuscript. PF and PT performed the field work and took care of children; RB, and PM contributed to data collection; FR carried out the paediatric diagnosis; FD, MT–K, RS, VL contributed to the study concept and design; VL, MT–K, FD obtained funding; VL first drafted the manuscript; Critical revision of the manuscript for important intellectual content: JH, RB, SB, VL, RS. All authors read and approved the final manuscript.

## Pre-publication history

The pre-publication history for this paper can be accessed here:



## Supplementary Material

Additional file 1 Tables_BMC-Public-Health-2008 Click here for file
