# Supplementary material for: 18-month occurrence of severe events among early diagnosed HIV-infected children before antiretroviral therapy in Abidjan, Côte d'Ivoire: A cohort study
Source: BMC Public Health. 2008 May 20;8:169. doi: 10.1186/1471-2458-8-169 (PMC2416449; doi:10.1186/1471-2458-8-169)
Supplement: Additional file 1 — Tables_BMC-Public-Health-2008 [file 1471-2458-8-169-S1.doc]

**18-month occurrence of severe events among early diagnosed HIV-infected children before antiretroviral therapy in Abidjan, Côte d’Ivoire: a cohort study**

**Table 1: Study characteristics of the ANRS049a DITRAME and ANRS 1201/1202 DITRAME-PLUS projects. Abidjan, Côte d’Ivoire. 1995-2005**

|  | **ANRS 049a DITRAME** | **ANRS 1201/1202 DITRAME PLUS** |
| --- | --- | --- |
| Study period | 1995-2000 | 2001-2005 |
| Study design | Randomized clinical trial and its open cohort | Prospective open-label cohort |
| Sites | Two districts of Abidjan | Two districts of Abidjan |
| Population | HIV-1 infected pregnant women >18 years,  36 weeks of gestation | HIV-1 infected pregnant women >18 years,  32-36 weeks of gestation |
| Maternal short-course (sc) antiretroviral peri-partum PMTCT intervention | Oral scZDV daily up to delivery  or placebo | Oral scZDV or scZDV+3TC daily up to delivery and sdNVP at beginning of labour. The scZDV+3TC maternal regimen was continued for three days post-partum |
| Neonatal PMTCT prophylaxis | None | One week ZDV + NVPsd at Day 2 |
| Postnatal PMTCT intervention on infant feeding | None | Formula feeding or  4-month shortened exclusive breastfeeding |
| Postnatal intervention | Standard care oriented on HIV-related clinical signs and symptoms | Online HIV-diagnosis at 6-week  Cotrimoxazole prophylaxis for infected children (25mg/kg/d)  Hemophilus influenzae B immunization |
| Follow-up procedures | Birth, Day2, Week 1, 6, M3, M6, M9, M12, M15, M18 | Birth, Day2, Week 1, 2, 3, 4, 6, M3, M4, M5, M6, M7, M8 M9, M12, M15, M18 |
| Outcomes | Hospitalization morbidity  Mortality | Severe morbidity collected on day-care hospital and Hospitalization morbidity  Mortality |

sdNVP : single dose nevirapine. PMTCT : Prevent HIV Mother-To-Child Transmission.

**Table 2: Baseline and follow-up characteristics of the 98 mothers and their peripartum HIV-infected children followed in the ANRS 049a DITRAME and ANRS 1201/1202 DITRAME-PLUS projects. Abidjan, Côte d’Ivoire. 1995-2005**

|  | ANRS 049a  DITRAME  (1995-2000)  N=53 | ANRS 1201/1202 DITRAME PLUS  (2001-2005)  N=45 | p-value |
| --- | --- | --- | --- |
| **Mothers characteristics** |  |  |  |
| Mean age at delivery (years; SE) | 26.6 (5.3) | 27.9 (5.9) | 0.25 |
| Mean parity (SE) | 2.1 (2.0) | 1.4 (1.8) | 0.08 |
| Education, n (%) |  |  | 0.63 |
| No schooling | 16 (41.0) | 14 (31.1) |  |
| Primary school | 9 (23.1) | 12 (26.7) |  |
| Secondary school or higher | 14 (35.9) | 19 (42.2) |  |
| WHO clinical staging 3-4, n (%) | 9 (17.0) | 18 (40.0) | 0.01 |
| Mean log_10_ HIV RNA viral load (SE) | 4.7 (0.6) | 5.0 (0.6) | 0.02 |
| Median lymphocyte CD4+ cell count x 10^6^/L (range) | 410 (41-1355) | 245 (45 - 1508) | 0.001 |
| < 200 CD4+ cell count x 10^6^/L, n (%) | 11 (20.8) | 17 (37.8) | 0.001 |
| **Infants characteristics** |  |  |  |
| Male, n (%) | 22 (41.5) | 20 (44.4) | 0.83 |
| Birth-weight (g., SE) | 2790 (490) | 2790 (520) | 0.95 |
| Birth-weight < 2500g, n (%) | 14 (26.4) | 12 (26.6) | 0.97 |
| Breastfed, n (%) | 51 (96.2) | 20 (44.4) | 0.001 |
| Mean log_10_ HIV RNA viral load peak (range) | 5.9 (4.4 – 7.3) | 6.3 (4.7 – 8.0) | 0.04 |
| Timing of infection, n (%) |  |  | 0.001 |
| In-utero infection | 7 (13) | 21 (47) |  |
| Intra-partum/early postnatal infection | 46 (87) | 24 (53) |  |
| Median follow-up duration (months, range) | 8.7 (0.4-18.0) | 12.1 (0.7-18.0) | 0.13 |
| Lost-to-follow-up, n (%) | 5 (9.4) | 2 (4.4) | 0.42 |
| Severe event before 18 months, n (%) | 38 (71.7) | 26 (57.7) | 0.15 |
| Death before 18 months, n (%) | 34 (64.1) | 23 (51.1) | 0.19 |
| Median age at death (months, range) | 5.7 (1.8 - 16.5) | 6.6 (0.7 -14.6) | 0.36 |

SE: standard error.

Table 3. Determinants of 18-month occurrence of severe events (hospitalization or death) (Cox proportional hazard model).

ANRS 1201/1202 Ditrame-Plus and ANRS 049a DITRAME projects, Abidjan, Côte d’Ivoire. 1995-2005

| Variables | Crude (Univariate) | | | Adjusted (multivariable) * | | |
| --- | --- | --- | --- | --- | --- | --- |
|  | HR# | 95% CI | p^§§^ | aHR | 95% CI | p |
| Cohort |  |  |  |  |  |  |
| DITRAME (reference) | 1 | - |  | 1 | - |  |
| DITRAME-PLUS (cotrimoxazole package) | 0.83 | 0.50-1.37 | 0.47 | 0.55 | 0.28 – 1.06 | 0.07 |
| Sex female (ref: male) | 1.29 | 0.78-2.13 | 0.31 | - | - | - |
| Low birth-weight (<2500g) | 1.33 | 0.79-2.28 | 0.29 | 1.39 | 0.80-2.43 | 0.24 |
| Ever breastfed (vs. not breastfed) | 0.99 | 0?57-1.73 | 0.98 | 0.58 | 0.28-1.22 | 0.15 |
| Child plasma viral load at 6-week  (for one log increase) | 1.11 | 0.81-1.52 | 0.48 | 1.19 | 0.83-1.70 | 0.33 |
| Maternal age <25 years (versus > 25 years) | 0.84 | 0.48-1.48 | 0.56 | - | - | - |
| Primigravida | 0.85 | 0.33-1.80 | 0.60 | - | - | - |
| Maternal WHO clinical HIV stage 3 or 4 (vs. 1 or 2) | 2.19 | 1.02-4.69 | 0.04 | 1.23 | 0.69-2.17 | 0.47 |
| Maternal viral load at inclusion (for one log increase) | 1.07 | 0.69-1.66 | 0.74 | - | - | - |
| Maternal CD4 count at inclusion (for 100 cells/µL) | 0.91 | 0.82-1.02 | 0.10 | - | - | - |
| Maternal death | 2.37 | 0.86-6.55 | 0.09 | 3.73 | 1.24 – 11.18 | 0.01 |

*Variables with p<0.25 in the univariate analysis; CI confidence interval;

^#^ univariate = unadjusted Cox proportional hazards model; HR: hazard ratio ; aHR: adjusted Hazard Ratio.

**Table 4. Probable causes of death** **(N=57) in the ANRS 049a DITRAME and ANRS 1201/1202 DITRAME-PLUS projects. Abidjan, Côte d’Ivoire. 1995-2005**

|  | DITRAME  N=34/53 | DITRAME-PLUS N=23/45 | p-value |
| --- | --- | --- | --- |
| Pneumonia, n (%) | 14 (41.2) | 4 (17.4) | 0.06 |
| Diarrhea, n (%) | 6 (17.6) | 10 (43.5) | 0.03 |
| Neurological disorders, n (%) | 3 (8.8) | 3 (13.0) | 0.46 |
| Failure-to-thrive, n (%) | 7 (20.6) | 4 (17.4) | 0.52 |
| Indeterminate, n (%) | 4 (11.8) | 2 (8.7) | 0.53 |
